# Lack of association between the *GRP78 *polymorphisms in the promoter and 3' UTR and susceptibility to chronic HBV infection in a Chinese Han population

**DOI:** 10.1186/1471-2350-11-83

**Published:** 2010-06-02

**Authors:** Xiao Zhu, Dong-Pei Li, Wen-Guo Fan, Marie CM Lin, Jin-Long Wang, Sheng-Qu Lin, Jian-Qing Huang, Hsiang-Fu Kung

**Affiliations:** 1 Institute of Oncology, Affiliated Tumor Hospital, Guangzhou Medical College, Guangzhou, PR China; 2 Zhongshan School of Medicine, Sun Yat-sen University, Guangzhou, PR China; 3 The Brain Tumor Center and Neurosurgery Division, Prince of Wales Hospital, The Chinese University of Hong Kong, Shatin, Hong Kong, PR China; 4 Stanley Ho Center for Emerging Infectious Diseases, and Li Ka Shing Institute of Medical Sciences, The Chinese University of Hong Kong, Hong Kong, PR China; 5 Open Laboratory of Chemical Biology, Institute of Molecular Technology, Department of Chemistry, The University of Hong Kong, Pokfulam, Hong Kong, PR China

## Abstract

**Background:**

Hepatitis B virus (HBV) infection causes large amount of unfolding or false-folding protein accumulation in the endoplasmic reticulum (ER), which in turn induces the expression of glucose-regulated protein 78 (GRP78). The aim in the present study was to analyse the potential association between GRP78 single-nucleotide polymorphisms (SNPs) and the risk of HBV infection.

**Methods:**

The associations between seven common *GRP78 *polymorphisms in the promoter (rs391957, rs17840762, rs17840761, rs11355458) and in the 3' untranslated region (UTR) (rs16927997, rs1140763, rs12009) and possible risk of chronic HBV infection were assessed in a case-control study. 496 cases and 539 individually matched healthy controls were genotyped.

**Results:**

Overall, no associations were observed in genotypic analyses. In addition, haplotypes and diplotypes combining those SNPs in the promoter or in the 3' UTR in high linkage disequilibrium (LD) were also not associated with HBV risk.

**Conclusion:**

These observations do not support a role for *GRP78 *polymorphisms in HBV infection in a predominantly Chinese Han population.

## Background

Hepatitis B virus (HBV) infection is one of the major infectious diseases that may lead to chronic liver disease, cirrhosis, and hepatocellular carcinoma (HCC) [1]. Approximately 30% of the world's population has been infected with HBV and approximately 350 million are persistent carriers. The mechanisms underlying resolution of acute HBV infection or its progression to chronicity remain undetermined [2]. Approximately 90% of infants infected from HBV e antigen (HBeAg) positive mothers will fail to achieve clearance and develop persistent HBV infection. Whereas, for adults, the majority of HBV-infected individuals achieve clearance with just less than 10% becoming persistent carriers[3].

Glucose-regulated protein 78 (GRP78), also recognized as immunoglobulin heavy-chain binding protein (BiP) as it was found bound to immunoglobulin heavy chains in pre-B cells, is known to be induced in the endoplasmic reticulum (ER) compartment by a variety of stresses [4]. These stress signals include glucose starvation, ER Ca2+ depletion, virus infection or cancer progress which leads to accumulation of misfolded proteins in the ER. It has been shown that GRP78 functions as an antiapoptotic chaperone playing a key role in maintaining the proper functions of proteins and organelles [5].

Recent studies have emphasized that *GRP78 *play important roles in the progression inhibition of chronic HBV infection and HCC progression [6,7]. Overexpression of the large superficial protein of HBV which translated from transcripts specified by a preS1 promoter in Huh7 cells results in a blockage of secretion of hepatitis B surface antigen (HBsAg), which leads to an accumulation of HBsAg in the ER lumen and in turn induces expression of GRP78[8]. The presence of pre-S mutants of HBV in sera and tissues was related to a high risk of developing HCC, and subsequently induce ER stress, leading to the expression of GRP78 [9-11].

In the previous projects, we focused on the possible association between HBV infection or HCC risk/prognosis and *GRP78 *polymorphism involving a new mutation (-87 T>A, from the estimated translation start site of *GRP78 *gene), or an intronic mutation (rs430397 G>A, in the intron 5 of *GRP78 *gene). These studies indicated that *GRP78 *was associated with HCC progression, but not associated with HBV infection [12,13]. As a part of series of studies about relationship of polymorphism(s) and stress-associated diseases, we hypothesized that polymorphisms in promoter and 3' untranslated region (UTR), which were known to be involved in regulation of gene expression, may be contributing factors or markers of a stress-associated disease, such as virus infection. To test this possibility, we further compared *GRP78 *polymorphisms at seven common loci in the promoter region (rs391957, rs17840762, rs17840761, rs11355458) and the 3' UTR (rs16927997, rs1140763, rs12009) between chronic HBV carriers and healthy subjects, and to reveal the possible association between HBV infection and *GRP78 *gene.

## Methods

### Subjects

496 Han patients with chronic HBV infection as case series who had been treated as inpatients or outpatients were enrolled in several affiliated hospitals of Guangzhou Medical College between January 2001 and April 2005. The diagnosis of chronic HBV infection was based on the presence of HBsAg and HBeAg, or HBsAg and anti-HBe, together with the absence of anti-HBs, for at least 36 months (according to the corresponding inpatient and outpatient records) prior to enrolment. All these were tested to exclude hepatitis C virus (HCV) or human immunodeficiency virus (HIV) infection, the presence of autoimmune hepatitis, and/or alcohol consumption exceeding 8 g per day and other conspicuous diseases. Patients who received antiviral therapy including interferon or nucleoside analogues within one year of enrollment were also excluded.

539 unrelated healthy HBV non-infected controls were recruited from Han volunteer blood donors in above hospitals and the Department of Cancer, Zhongshan School of Medicine in Sun Yat-sen University in the same time. The controls were negative for HBsAg, anti-HBs and anti-HBc, with normal serum alanine aminotransferase (ALT) levels. These subjects were members of the health-care staff of the hospitals who were periodically monitored for professional exposure to hepatitis viruses. A standard informed consent, was given to the participants, after the nature of study had been fully explained. The study was performed with the approval of the ethical committee of Guangzhou Medical College and adhered to the tenets of the Declaration of Helsinki.

### Genotyping

Genomic DNA was extracted from peripheral blood leukocytes using QIAGEN QIAamp DNA Mini Blood Kit (Hilden, Germany). The SNPs were detected by the TaqMan Assay-by-Design service (Applied Biosystems, Foster City, CA). The details of sequences and reaction conditions are available upon request https://products.appliedbiosystems.com/ab/en/US/adirect/ab. PCR was performed using the TaqMan Universal Master Mix without UNG on the ABI PRISM 7900 HT Sequence Detection System (Applied Biosystems, Foster City, CA) and heated to 95°C for 10 minutes followed by 40 cycles of 92°C for 15 seconds and 60°C for 1 minute.

### Haplotype and diplotype construction

Haplotype frequencies and pairwise linkage disequilibrium (LD) matrices were determined using the Haploview version 3.2.0 (Whitehead Institute for Biomedical Research, USA)[14]. Magnitudes of LD were represented by the standardized LD coefficients, complete association (D') and absolute association (r^2^) [15-17]. The frequencies closely agreed with results from a maximum likelihood method implemented via an expectation-maximization (EM) algorithm[18]. Haplotypes and diplotypes were selected according to the corresponding occurring probabilities with a higher likelihood (> 0.95 as cut-point) [19].

### Statistical analysis

Chi-square test was used to determine whether there is a significant difference between cases and controls in terms of gender. Mann-Whitney U-test was used to test the difference among the age groups. Hardy-Weinberg equilibrium (HWE) of genotype distribution among cases or controls was carried out using Pearson Chi-square test. Logistic regression analysis was used to estimate odds ratios (ORs) and corresponding 95% confidence intervals (CIs) comparing cases to controls in association with genotypes, haplotypes and diplotypes using dominant, recessive and co-dominant models, respectively, adjusted for age and sex. All statistical tests were 2-sided and statistical significance was taken as *P *value less than 0.05.

## Results

In the cases, the age at diagnosis ranged 25.5-59.8 years, the mean age was 41.9 (± 13.5) years, and the gender (male/female) ratio was 1.57:1. In the controls, the age ranged 27.0-56.2 years, the mean age was 43.0 (± 9.7) years, and the gender ratio was 1.32:1. The cases and the controls were frequency-matched by age (± 5 years, *p *= 0.130) and gender (*p *= 0.177), but not by serum ALT levels (*p *= 0.008) (Table 1).

**Table 1:** Characterization of the participants

Characteristics	**Cases **(%)	**Controls **(%)	*P*
n	496	539	
Age (mean ± SD)	41.9 ± 13.5	43.0 ± 9.7	0.130^a^
Gender			
Females	193 (38.91)	232 (43.04)	
Males	303 (61.09)	307 (56.96)	0.177^b^
ALT (U/L, means ± SD)	147.1 ± 95.5	26.7 ± 11.4	0.008^a^
HBsAg (+)	496 (100%)	0	
anti-HBs (+)	0	0	
HBeAg (+)	193 (38.91)	0	
anti-HBe (+)	303 (61.09)	0	

Abbreviations: SD, standard deviation; ALT, alanine aminotransferase.

^a^Mann-Whitney test; ^b^Pearson Chi-square test.

Genotyping data for each single-nucleotide polymorphism (SNP) were successfully obtained for 100% of the subjects. The distribution of the genotypes in controls and cases did not deviate from that expected by Hardy-Weinberg equilibrium (data not shown). Table 2 showed the distribution of genotypic frequencies of the 7 SNPs in chronic HBV patients and control subjects. No statistically significant differences were found between cases and controls in any of the dominant, codominant or recessive genotype models as well as the presence of single variant alleles (*p*-trend > 0.05, respectively).

**Table 2:** Genotype frequencies of *GRP78 *among cases and controls, and risk of HBV

Loci	Genotypes	Cases (%)	Controls (%)	OR (95% CI)	*P*
rs391957	GG	275 (54.95)	320 (59.22)	1	
	AG	198 (40.00)	200 (37.24)	1.22 (0.94-1.59)	0.137
	AA	23 (5.05)	19 (3.54)	1.60 (0.84-3.07)	0.354
*p*-trend^a^				0.106	
rs17840762	CC	334 (67.39)	366 (67.97)	1	
	CT	144 (29.19)	151 (27.93)	1.05 (0.79-1.39)	0.659
	TT	18 (3.42)	22 (4.10)	0.78 (0.41-1.51)	0.271
*p*-trend^a^				0.501	
rs17840761	TT	130 (25.76)	156 (28.86)	1	
	CT	236 (47.74)	249 (46.37)	1.17 (0.87-1.59)	0.405
	CC	130 (26.48)	134 (24.77)	1.24 (0.88-1.75)	0.222
*p*-trend^a^				0.245	
rs11355458	d d	275 (54.95)	320 (59.22)	1	
	d G	198 (40.00)	200 (37.24)	1.22 (0.94-1.59)	0.137
	GG	23 (5.05)	19 (3.54)	1.60 (0.84-3.07)	0.354
*p*-trend^a^				0.106	
rs16927997	TT	440 (88.83)	480 (89.01)	1	
	CT	52 (10.45)	55 (10.24)	1.01 (0.67-1.53)	0.812
	CC	4 (0.72)	4 (0.74)	1.04 (0.23-4.68)	0.460
*p*-trend^a^				0.393	
rs1140763	TT	129 (25.40)	168 (31.10)	1	
	CT	236 (48.29)	246 (45.62)	1.36 (0.92-1.79)	0.348
	CC	131 (26.31)	125 (23.28)	1.51 (0.95-2.06)	0.177
*p*-trend^a^				0.204	
rs12009	TT	166 (31.61)	204 (37.80)	1	
	CT	222 (45.59)	237 (43.95)	1.25 (0.88-1.61)	0.291
	CC	108 (21.80)	98 (18.25)	1.53 (0.96-2.08)	0.097
*p*-trend^a^				0.082	

Note: OR, odds ratio; d, delete base.

^a^Test for trend of odds was two sided and was based on likelihood ratio tests assuming a multiplicative model for all cases.

**Figure 1:**
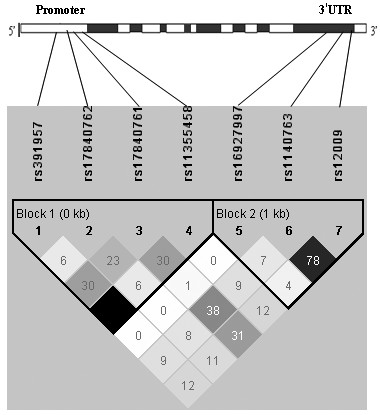
**Linkage disequilibrium (LD) pattern (r^2 ^plots) of the SNPs in the promoter and 3' UTR of *GRP78 *gene among the 496 chronic HBV carriers, as generated by Haploview v3.2**. Coding exons are marked by shaded blocks. The blocks are designed according to the internally developed solid spine of LD. The value within each diamond represents the pairwise correlation between pairs of SNPs (measured as 100 × r^2^) defined by the upper left and the upper right sides of the diamond. The diamond without a number corresponds to r^2 ^= 1.

LD reflects the non random association of alleles at two or more loci. Pair-wise LD analysis in 439 chronic HBV carriers showed the two blocks (block 1, promoter, including rs391957, rs17840762, rs17840761 and rs11355458; Block 2, 3' UTR, including rs16927997, rs1140763 and rs12009) which are designed according to the internally developed solid spine of LD (Figure 1). Rs391957 and rs11355458 were completely linked (r^2 ^= 1.00) and rs1140763 and rs12009 were in high linkage disequilibrium (r^2 ^= 0.785) in these carriers.

Because genetic effects are not exerted individually, we considered the combined effects of the all SNPs in this study according to the LD. There are 4 haplotypes and 9 diplotypes in the promoter region, and 3 haplotypes and 6 diplotypes in the 3' UTR were constructed based on the 7 SNPs with minor allelic frequencies of above 1% in the chronic HBV carriers. The distributions of haplotypes and diplotypes exhibited no significant differences from the risk of HBV infection (*p*-trend > 0.05, respectively, Table 3 and Table 4).

**Table 3:** Haplotype frequencies in the promoter region and the 3' UTR of *GRP78 *gene in HBV carriers and controls, and risk of HBV

Haplotypes	Cases (%)	Controls (%)	OR (95% CI)	*P*
Promoter				
G-C-T-d	496 (49.64)	561 (51.96)	1	
A-C-C-G	244 (25.04)	238 (22.16)	1.26 (0.92-1.58)	0.107
G-T-C-d	180 (18.02)	195 (18.08)	1.05 (0.80-1.35)	0.376
G-C-C-d	72 (7.30)	84 (7.82)	0.95 (0.71-1.32)	0.552
*p*-trend^a^			0.247	
3' UTR				
T-T-T	494 (49.55)	582 (53.91)	1	
T-C-C	438 (44.50)	433 (40.22)	1.15 (0.87-1.46)	0.269
C-C-T	60 (5.95)	63 (5.87)	1.21 (0.76-1.65)	0.122
*p*-trend^a^			0.181	

Note: OR, odds ratio, d, delete base.

^a^Test for trend of odds was two sided and was based on likelihood ratio tests assuming a multiplicative model for all cases.

**Table 4:** Diplotype frequencies in the promoter region and the 3' UTR of *GRP78 *gene in HBV carriers and controls, and risk of HBV

Diplotypes	**Cases **(%)	Controls (%)	OR (95% CI)	*p*
Promoter				
GCTd/GCTd	130 (25.77)	156 (28.86)	1	
ACCG/GCTd	123 (24.86)	128 (23.84)	1.20 (0.83-1.76)	0.196
GCTd/GTCd	75 (15.14)	81 (14.90)	1.11 (0.74-1.65)	0.153
ACCG/GTCd	55 (11.17)	49 (9.12)	1.57 (0.97-2.55)	0.462
GCCd/GCTd	38 (7.75)	40 (7.45)	1.19 (0.72-2.07)	0.259
ACCG/GCCd	20 (3.96)	23 (4.28)	1.04 (0.53-2.00)	0.754
GTCd/GTCd	18 (3.42)	22 (4.10)	0.89 (0.45-1.75)	0.410
GCCd/GTCd	14 (2.88)	21 (3.91)	0.84 (0.41-1.72)	0.388
ACCG/ACCG	23 (5.05)	19 (3.54)	1.66 (0.85-3.24)	0.071
*p*-trend^a^			0.142	
3' UTR				
TCC/TTT	203 (41.62)	214 (39.66)	1	
TTT/TTT	129 (25.41)	168 (31.10)	1.30 (0.95-1.80)	0.249
TCC/TCC	108 (21.80)	98 (18.25)	1.50 (0.75-2.28)	0.166
CCT/TTT	33 (6.67)	32 (5.96)	1.43 (0.82-2.46)	0.215
CCT/TCC	19 (3.78)	23 (4.28)	0.97 (0.55-2.13)	0.647
CCT/CCT	4 (0.72)	4 (0.74)	1.36 (0.26-6.08)	0.076
*p*-trend^a^			0.091	

Note: OR, odds ratio; d, delete base.

^a^Test for trend of odds was two sided and was based on likelihood ratio tests assuming a multiplicative model for all cases.

## Discussion

This was one of the series of studies to investigate the association between *GRP78 *polymorphisms and risk of HBV infection. In this case-control study, there were no significant differences in risk of HBV associated with SNPs at any of these loci in the promoter and the 3' UTR of *GRP78 *gene. A single SNP usually provides a little information. If more SNPs are unified to construct haplotypes and diplotypes, they would supply more information and make up for short-coming of single SNP [20-22]. The SNPs identified in this study comprised 4 haplotypes and 9 diplotypes in the promoter region, and 3 haplotypes and 6 diplotypes in the 3' UTR. By analyzing the associations between haplotypes/diplotypes and chronic HBV infection, however, no association were found.

The *GRP78 *promoter contains three ER stress response elements (ERSEs) consisting of a tripartite structure CCAATN9CCACG, with N being 9-bp GC-rich region [23]. The four SNPs examined located not within but upstream of ERSE. *GRP78 *promoter haplotypes may affect the individual variability of ER stress response and has been reported to be a potential risk factor for bipolar disorder in a Japanese population [24]. These suggested that the promoter haplotypes in the *GRP78 *gene were significantly associated with the functional SNPs, which were involved in the promoter activity. However, our study did not demonstrate an association between the promoter haplotypes and diplotypes with chronic HBV infection in our Chinese population.

The rs16927997 is located next to the ATTTA motif and substitution from T to C reduces the contents of AT. Around the rs16927997, there are four ATTTA motifs with AT-rich contents, which suggest that the region is so-called "AU-rich element". Although AU-rich element is associated with mRNA unstability and promotes degradation of mRNA in most cases [25,26], significant difference was not observed between rs16927997-T and rs16927997-C in a mRNA degradation assay [24]. The present study further suggested that the alleles and genotypes of rs16927997, rs1140763 and rs12009 in the 3' UTR of *GRP78 *gene, including the corresponding haplotypes and diplotypes, were not associated with the risk of chronic HBV infection.

A number of contingent environmental conditions, among which infectious diseases may have been the most powerful, have exerted variable pressures on the human genome and favored the selection of alleles interfering with disease physiopathology. GRP78 has recently emerged as an intracellular antiviral factor against HBV [6]. HBV invasion and other physiopathologic changes cause large amount of unfolding or false-folding protein accumulation in the ER, which in turn induces expression of GRP78 [27]. GRP78 pathway was one of the most important responders to virus-associated stress [28,29]. But the mechanism was controversial. Possibly, GRP78 enhance primary immune protection against HBV. But its polymorphisms do not play more important roles during the chronic inflammation process. However, additional studies from larger populations among Han Chinese, and from diverse ethnic populations, are warranted before the importance of *GRP78 *polymorphisms in chronic HBV risk can be fully ascertained.

## Conclusion

In conclusion, the present study suggests that the *GRP78 *polymorphisms are not associated with chronic HBV infection. These data, however, do not exclude a possible physiopathological role of these SNPs in the progression of chronic HBV infection.

## Competing interests

The authors declare that they have no competing interests.

## Authors' contributions

ZX and LDP participated of the study design and performed the genotyping. FWG and HJQ performed the statistical analysis. WJL and LSQ participated in coordination and sample collection. LMCM and KHF drafted the manuscript. All authors read and approved the final manuscript.

## Pre-publication history

The pre-publication history for this paper can be accessed here:

http://www.biomedcentral.com/1471-2350/11/83/prepub
